# The role of vitamin D in gastrointestinal cancer prevention and management: a narrative review

**DOI:** 10.1097/MS9.0000000000004114

**Published:** 2025-10-16

**Authors:** Nasar Nasrullah Khan, Tusneem Shehzadi, Zim Warda Hasan, Ariana Panezai, Muhammad Naqash, Asad Ur Rehman, Anmol Mohan, Dev Tanush, Hasiba Karimi, Hasibullah Aminpoor, Usha Tejwaney, Sarwan Kumar

**Affiliations:** a Department of Medicine, Nottingham University Hospital, Nottingham, UK; b Department of Medicine, University College of Medicine and Dentistry, Karachi, Pakistan; c Clinical and Translational Science, School of Medicine, West Virginia University, Morgantown, WV, USA; d Department of Medicine, Ziauddin Medical College, Karachi, Pakistan; e Department of Internal Medicine, Hull Royal Infirmary Hull, Hull, UK; f Independent Medical College Faisalabad, Faisalabad, Punjab, Pakistan; g Department of Medicine, Mayo Clinic, Rochester, MN, USA; h Department of Medicine, Ziauddin Medical University, Karachi, Pakistan; i Bezmialem Vakif University, Faculty of Medicine, Istanbul, Turkey; j Kabul University of Medical Sciences “Abu Ali Ibn Sina”, Kabul, Afghanistan; k Valley Health System, Paramus, New Jersey, USA; l Wayne State University, Detroit, Michigan, USA

**Keywords:** cancer prevention and management, gastrointestinal cancer, vitamin D

## Abstract

Gastrointestinal (GI) cancers, including colorectal, gastric, esophageal, pancreatic, and liver cancers, represent a significant global health burden, with high prevalence and mortality rates driven by aging, lifestyle factors, and infections. Emerging research highlights the role of vitamin D in cancer biology, particularly its potential in the prevention and management of GI cancers. Through its active form, calcitriol, vitamin D regulates cell proliferation, apoptosis, angiogenesis, and immune responses via the vitamin D receptor (VDR). Epidemiological studies suggest an inverse association between vitamin D deficiency and cancer risk, while experimental models demonstrate its anti-tumor effects. Additionally, vitamin D influences gut microbiota composition and immune regulation, potentially enhancing cancer immunity and response to immunotherapy. Despite promising evidence, clinical trials have yielded mixed results, underscoring the need for further research to determine optimal supplementation strategies, individual responses, and genetic influences. This review explores vitamin D’s mechanisms, epidemiological evidence, and therapeutic potential in GI cancers, advocating for personalized approaches in cancer prevention and treatment.

## Introduction

Gastrointestinal (GI) cancers—including colorectal, gastric, esophageal, pancreatic, and liver cancers—represent a major global health challenge, driven by aging populations, lifestyle factors, and chronic infections such as *Helicobacter pylori* and hepatitis viruses^[1,2]^. The gut microbiota contributes to GI carcinogenesis through modulation of inflammation and immune responses^[3]^. Advances in molecular biology, genetics, and imaging have improved early detection, diagnosis, and treatment strategies^[4]^; however, the burden remains disproportionately high in Asia due to limited healthcare access and screening infrastructure^[2]^. Cost-effective prevention strategies—including vaccination, public education, and targeted screening—are critical to reducing this burden^[2,3]^. Globally, GI cancers account for approximately 26% of all cancer cases and 35% of cancer-related deaths, with geographic variation in prevalence: esophageal, gastric, and liver cancers are more frequent in Asia, whereas colorectal and pancreatic cancers predominate in Europe and North America^[5,6]^. Key modifiable risk factors include tobacco use, alcohol consumption, obesity, and chronic infections (e.g., *H. pylori*, HBV, HCV), while systemic conditions such as diabetes and hypertension may contribute via oxidative stress and chronic inflammation^[7]^. Prevention, screening, and lifestyle interventions remain central to reducing GI cancer incidence and mortality^[2,5]^.

**HIGHLIGHTS**

- This paper highlights the inverse correlation between vitamin D levels and gastrointestinal cancer risk, with observational studies suggesting a protective effect, though confounding factors complicate causal inference.
- This paper discusses the mixed outcomes of vitamin D supplementation trials in cancer prevention and treatment, indicating potential benefits in specific subgroups but lacking consistent evidence for widespread use.
- This paper highlights the role of genetic and epigenetic factors in modifying vitamin D’s impact on cancer, underscoring the need for precision medicine approaches to optimize prevention and therapeutic strategies.

Vitamin D, traditionally recognized for its role in calcium homeostasis and bone metabolism, also modulates immune function by regulating both innate and adaptive immunity^[8–10]^. Through binding to the vitamin D receptor (VDR), it is locally converted to its active form, 1,25-dihydroxyvitamin D3, suppressing autoimmune responses and inflammatory cytokine production^[11]^. Deficiency has been associated with autoimmune and inflammatory disorders, including diabetes, asthma, and rheumatoid arthritis^[10]^. Despite these mechanistic insights, evidence for therapeutic use in immune-inflammatory or hyperproliferative disorders remains limited^[8]^. Vitamin D deficiency is prevalent worldwide, affecting populations across all age groups, including those in sunny or industrialized regions^[12,13]^. Prevalence is especially high in the Middle East, with limited data from South America and Africa^[12,13]^. Deficiency contributes to rickets, osteoporosis, and may increase risk for chronic diseases including diabetes, hypertension, and cancers^[14,15]^. Risk factors include low sunlight exposure, aging, and inadequate dietary intake^[14]^. Experts recommend maintaining serum 25-hydroxyvitamin D levels above 75 nmol/L through adequate sun exposure and supplementation (800–1000 IU/day)^[15]^.

Emerging evidence suggests that vitamin D may influence GI cancer risk by regulating cellular proliferation, differentiation, and apoptosis via calcitriol and VDR-mediated pathways^[16–19]^. However, clinical trial data remain inconsistent, potentially due to differences in study design, dosage, baseline vitamin D status, and genetic variability in VDR polymorphisms^[16–19]^. This highlights the need to critically evaluate both observational and interventional evidence, considering the hierarchy of study designs to inform translational and clinical applications. We confirm that no AI tools were utilized in the conduct or writing of this manuscript, in accordance with the TITAN checklist^[20]^.

## Mechanisms of vitamin D in cancer biology

Vitamin D, particularly its active metabolite calcitriol (1,25-dihydroxyvitamin D3), exerts anticancer effects via multiple cellular pathways. Binding to the vitamin D receptor (VDR), calcitriol regulates gene expression involved in proliferation, differentiation, apoptosis, and DNA repair^[21–24]^. It also inhibits tumor progression through suppression of angiogenesis, metastasis, and inflammatory pathways, including NF-κB signaling and prostaglandin metabolism^[23]^. Notably, calcitriol modulates over 200 genes associated with immune function and cellular stress responses, which may be relevant to tumorigenesis^[24]^.

The VDR plays a central role in gastrointestinal (GI) homeostasis, influencing epithelial proliferation, differentiation, barrier integrity, and mucosal immunity^[25–27]^. VDR expression declines progressively from normal to premalignant to malignant tissues in gastric cancer, suggesting prognostic relevance^[25]^. Colorectal tumors show variable VDR expression, often higher than adjacent normal tissue, underscoring tissue-specific regulatory roles^[26]^. Chronic intestinal inflammation, a recognized risk factor for colon cancer, is associated with impaired VDR signaling and diminished responsiveness to anti-inflammatory stimuli^[28]^.

Emerging evidence highlights interactions between vitamin D, the gut microbiota, and cancer immunity. Vitamin D regulates intestinal VDR expression, shapes microbiota composition, and modulates systemic inflammation^[29,30]^. Deficiency promotes dysbiosis and inflammation, predisposing to tumorigenesis^[31]^. In murine models, vitamin D supplementation enhances immune-mediated tumor resistance and improves responses to immune checkpoint inhibitors, partly by modulating epithelial cells to favor beneficial microbial species such as *Bacteroides fragilis*^[32]^. Human data link elevated vitamin D-induced gene expression with improved outcomes in checkpoint inhibitor therapy, supporting potential roles in immunomodulation^[32]^.

## Epidemiological evidence

Ecological studies consistently demonstrate inverse associations between solar ultraviolet B (UVB) exposure, population-level vitamin D status, and cancer incidence and mortality across diverse regions, including the United States, Australia, China, Japan, and Spain^[33,34]^. These associations have been documented for at least 15 cancer types, such as bladder, breast, colon, and lung cancers^[33]^. Geographic patterns, including variations in prostate cancer mortality, further support this protective link^[35]^. Multifactorial analyses adjusting for confounders like smoking, alcohol use, and urbanization still find a strong UVB–vitamin D–cancer relationship^[36]^. However, a critical limitation of ecological studies is the **ecological fallacy**—the assumption that group-level trends reflect individual-level causality, which may not hold true. Despite this, such studies provide important initial signals that merit further investigation through more rigorous designs.

Observational studies and meta-analyses exploring vitamin D status and gastrointestinal (GI) cancer risk have yielded mixed results. For gastric cancer, a meta-analysis by Khayatzadeh et al. (2015) reported no significant association between vitamin D intake or serum levels and cancer risk (pooled OR 0.94, 95% CI 0.83–1.07)^[37]^. In contrast, a pooled analysis of 17 prospective cohorts by McCullough et al. (2018) found that individuals with higher circulating 25(OH)D levels had a significantly lower risk of colorectal cancer, particularly among women (RR 0.68, 95% CI 0.53–0.87 for ≥75 vs. <30 nmol/L)^[38]^. For upper GI cancers, Abnet et al. (2010) observed no significant overall association, although subgroup analyses indicated variable effects based on race and smoking status^[39]^. A broader review by Sluyter et al. (2020) concluded that while vitamin D supplementation does not significantly reduce cancer incidence, it was associated with a 16% reduction in total cancer mortality (RR 0.84, 95% CI 0.74–0.95)^[40]^. These findings highlight the variability across cancer types and underscore the need to better understand population-specific effects and underlying mechanisms.

Establishing causality in vitamin D–cancer associations remains a challenge, particularly in observational studies where **confounding** and **reverse causation** are major concerns^[41,42]^. To address these limitations, researchers have employed advanced methodological tools such as discordant twin designs, negative controls, instrumental variables (e.g., Mendelian randomization), and natural experiments^[41,42]^. These approaches aim to approximate randomized conditions and strengthen causal inference when randomized controlled trials (RCTs) are infeasible. The **counterfactual model** provides a conceptual foundation for understanding these strategies and evaluating the validity of observed associations^[41,43]^. In gene–lifestyle interaction studies involving vitamin D, careful application of causal inference criteria (e.g., temporality, dose–response, biological plausibility) is essential^[44]^. While administrative and survey data remain useful sources of epidemiological insight, they require sophisticated statistical analysis to minimize bias and produce reliable estimates of effect^[43]^.

Together, these lines of evidence suggest that vitamin D status may play a meaningful role in the prevention and progression of gastrointestinal cancers. However, inconsistent findings across study designs, populations, and cancer types indicate that further high-quality RCTs, alongside robust causal inference strategies—are necessary to move from association to clinical application. The epidemiological data is summarized in Table 1.

**Table 1:** Summary of past studies

Section	Key findings	Study insights	Challenges identified	Research needs	References
Observational Studies	- Strong inverse correlation between UVB exposure, vitamin D levels, and cancer incidence/mortality.- Protective effect demonstrated for 15+ cancer types.	- Observed in the U.S., Australia, China, Japan, Spain, and global analyses.- Linked to cancer types like bladder, breast, colon, and lung.	- Multifactorial analyses confirm UVB-vitamin D-cancer links but highlight complex interactions with other risk factors.	- Further studies to isolate vitamin D’s effect from UVB exposure.- Exploration of treatment outcomes linked to vitamin D status.	^[33–36]^
Meta-Analyses and Systematic Reviews	- Mixed results for vitamin D status and gastrointestinal cancer outcomes.- Reduced colorectal cancer risk with higher 25(OH)D levels in women.	- Pooled analyses of 17 cohorts support an inverse association with colorectal cancer.- No significant link to gastric cancer risk.	- Limited benefits in randomized trials.- Subgroup differences (e.g., by race and smoking status) complicate interpretations.	- Well-designed, large-scale trials considering subgroup variations.- Comprehensive meta-analyses across diverse populations.	^[37–40]^
Confounding Factors and Limitations	- Observational studies struggle with causal inference due to confounders and reverse causation.	- Methods like discordant-twin designs and instrumental variables improve causal inference.	- Difficulties in gene-lifestyle interaction studies.- Biases in observational data from surveys and administrative records.	- Use of advanced statistical techniques to minimize bias.- Validation of observational findings through experimental or quasi-experimental designs.	^[41–44]^

## Clinical evidence and interventional studies

### Vitamin D supplementation trials

Randomized controlled trials (RCTs) assessing the impact of vitamin D supplementation on gastrointestinal (GI) cancer prevention have produced inconsistent results. The VITAL trial, a large double-blind RCT involving over 20,000 participants, investigated the effects of daily vitamin D_3_ (2000 IU) on cancer incidence. The study found no significant reduction in overall cancer incidence in the intervention group compared to placebo (Hazard Ratio [HR] 0.96; 95% Confidence Interval [CI], 0.88–1.06; p = 0.47)^[45]^. However, exploratory subgroup analyses suggested that individuals with lower baseline vitamin D levels might derive greater benefit. Critically, GI cancer prevention was a **secondary endpoint** in this trial, and the participants were largely vitamin D replete at baseline, which may have limited the observed effect. Additionally, the daily dose of 2000 IU may not have been sufficient to achieve protective serum 25(OH)D levels in all participants, particularly considering interindividual variability due to genetic polymorphisms in VDR and vitamin D metabolism genes.

A review of supplementation trials in elderly women similarly concluded that vitamin D had no clear preventive effect on cancer occurrence but may contribute to reduced cancer mortality^[46]^. Across studies, heterogeneity in dosing regimens, duration, participant selection, and vitamin D formulation, ranging from cholecalciferol to active analogs like calcitriol, complicates interpretation^[47]^. Low adherence, variations in baseline 25(OH)D levels, and inadequate stratification for factors such as BMI, sunlight exposure, and genetic variation may have influenced outcomes^[18,47]^. These findings emphasize the need for individualized trial designs that account for biological variability, baseline vitamin D status, and potential gene–environment interactions. These effects are summarized in Table 2.

**Table 2:** Vit D trials and their implications in cancer prevention

Section	Key findings	Study insights	Challenges identified	Research needs	References
Vitamin D Supplementation Trials	- Mixed results on vitamin D’s role in GI cancer prevention and treatment. —VITAL trial: Testing 2000 IU/day in 20,000 adults.	- No clear evidence of reduced cancer incidence but possible reduction in mortality.	- Variation in dosages (e.g., D3, calcitriol). —Compliance and baseline vitamin D levels impact results.	- Determination of optimal dosing, duration, and target populations. —Addressing variability in trial designs.	^[18,45–47]^
Effect on Cancer Prognosis	- Vitamin D supplementation showed a 30% reduction in adverse colorectal cancer outcomes (meta-analysis).	- AMATERASU trial: Subgroup benefits for 25(OH)D levels of 20–40 ng/mL. —High prevalence of deficiency (87.6%).	- No consistent improvement in relapse-free survival. —Insufficient evidence for routine supplementation in cancer care.	- Further randomized trials on specific subgroups. —Standardized protocols to evaluate baseline and supplementation impact.	^[48–50,54]^
Gaps in Clinical Research	- Inconsistent findings on vitamin D’s protective role in GI cancers. —Genetic variations affect response to supplementation.	- Subgroup differences observed based on race, smoking, and baseline levels.	- Lack of tailored approaches to supplementation. —Inadequate consideration of individual genetic and environmental factors.	- Targeted research on genetic and environmental interactions. —Focus on population-specific responses to supplementation and cancer risk reduction.	^[39,51–53]^

### Effect on cancer prognosis

Several studies have explored whether vitamin D supplementation improves outcomes in patients with established GI cancers. A meta-analysis of RCTs found a 30% relative reduction in adverse colorectal cancer outcomes associated with vitamin D supplementation (Relative Risk [RR] 0.70; 95% CI, 0.55–0.89)^[48]^. However, other trials have reported null results. The AMATERASU trial, which enrolled 417 patients with resected digestive tract cancers, found no significant difference in relapse-free survival between the vitamin D and placebo groups (HR 0.76; 95% CI, 0.50–1.14; p = 0.18). Subgroup analysis indicated potential benefit in patients with intermediate baseline 25(OH)D levels (20–40 ng/mL), but these findings were not conclusive. Notably, the trial did not incorporate genetic stratification, which could have influenced responsiveness due to VDR polymorphisms.

Vitamin D deficiency is prevalent among GI cancer patients, with one study reporting a deficiency rate of 87.6%, which improved significantly following supplementation^[49]^. Low serum vitamin D levels have been linked to poorer prognosis in several cancer types; however, a systematic review concluded that current evidence is insufficient to recommend routine vitamin D supplementation as part of cancer management^[50]^. The inconsistent findings highlight the complexity of vitamin D’s role in cancer progression, influenced by baseline vitamin D status, tumor heterogeneity, VDR expression, and potential gene environment interactions, underscoring the necessity for well-powered, biomarker-driven trials. These effects are summarized in Table 2.

### Gaps in clinical research

Despite considerable investigation, the role of vitamin D in GI cancer prevention and prognosis remains inconclusive. Variability in trial outcomes may be attributed to differences in population characteristics, baseline vitamin D status, and the methods used to assess and supplement vitamin D^[51]^. Importantly, genetic factors, including single nucleotide polymorphisms (SNPs) in genes related to vitamin D metabolism and VDR expression, may influence individual responsiveness to supplementation^[52]^. These polymorphisms are rarely considered in current trial designs, limiting their applicability across genetically diverse populations.

Moreover, many large-scale RCTs do not stratify participants based on baseline 25(OH)D levels or correct for confounding lifestyle factors such as physical activity and diet, which may mask potential effects^[53]^. Subgroup analyses have revealed that associations between vitamin D and cancer risk may differ by race, sex, or smoking status, particularly in upper GI cancers^[39]^. Taken together, these findings support a more tailored approach to future research one that incorporates baseline vitamin D assessment, genetic profiling (e.g., VDR and vitamin D metabolism SNPs), stratified randomization, and standardized protocols for measuring vitamin D exposure and bioavailability. Such precision approaches would help clarify true clinical benefit and identify responder subgroups in GI cancer prevention and prognosis.

## Molecular subtypes and personalized medicine

Vitamin D deficiency is common among patients with gastrointestinal (GI) cancers, with up to 87.6% of individuals demonstrating suboptimal serum 25(OH)D levels^[49]^. Epidemiological studies consistently associate low vitamin D levels with increased cancer risk and poorer prognosis^[16]^. Mechanistically, 1,25-dihydroxyvitamin D_3_ modulates key cellular pathways regulating proliferation, apoptosis, angiogenesis, and differentiation through its action on vitamin D receptor (VDR)-mediated gene expression^[16,18]^. Vitamin D also supports gut homeostasis by influencing epithelial integrity and immune regulation^[18]^. Supplementation has been shown to significantly reduce vitamin D deficiency prevalence in cancer patients, with rates declining from 91.3% to 57.6% following intervention^[49]^. Preclinical studies further indicate that vitamin D may inhibit cancer stem cell activity and associated signaling pathways across various GI malignancies, including esophageal, gastric, colorectal, pancreatic, and hepatic cancers^[55]^. These mechanistic insights provide a biological rationale for exploring vitamin D as a potential preventive and adjunctive therapeutic agent.

However, the effectiveness of vitamin D-based interventions is likely modulated by genetic and epigenetic variability, which may explain inconsistencies observed in clinical studies. Polymorphisms in the VDR gene, including FokI, BsmI, TaqI, and ApaI, have been implicated in cancer risk across multiple sites, though findings remain inconsistent^[56]^. These inconsistencies may be influenced by population-specific factors such as ethnicity, UVB exposure, and baseline vitamin D levels. Additionally, polymorphisms in genes involved in vitamin D metabolism, such as CYP27B1, CYP24A1, and the vitamin D-binding protein gene GC, alter serum vitamin D concentrations and modulate cancer risk profiles^[57]^. For example, the GC rs7041 TT genotype has been linked to elevated breast cancer risk, whereas the VDR FokI ff genotype confers protective effects in certain populations^[58]^. Emerging evidence in thyroid cancer suggests that these genetic determinants may exert a stronger predictive value than classical environmental factors, such as sun exposure or diet^[59]^.

Despite compelling preclinical data, large clinical trials have not consistently demonstrated a significant preventive benefit of vitamin D supplementation in cancer^[53]^. This discrepancy between mechanistic promise and clinical efficacy likely reflects high interindividual variability in vitamin D metabolism and response. Factors such as tumor heterogeneity, differential VDR expression across tumor subtypes, and local tumor microenvironmental influences, including immune infiltration and stromal interactions, likely contribute to these variable outcomes^[60,61]^. For instance, in colorectal cancer, VDR expression differs by tumor location and stage, potentially influencing responsiveness to supplementation. Furthermore, most RCTs, including those assessing GI cancer endpoints, did not stratify participants based on genetic polymorphisms or baseline vitamin D status, which may have masked potential benefits in susceptible subgroups.

To bridge these translational gaps, precision oncology approaches are increasingly advocated. These include integrating genetic and epigenetic profiling, tumor subtype classification, and circulating biomarkers to guide vitamin D-based interventions^[62]^. Non-invasive tools, such as liquid biopsy-derived cell-free DNA, exosomal microRNAs, and transcriptomic markers, offer promising avenues for assessing intratumour heterogeneity and stratifying patients for tailored treatment^[61,62]^. Ultimately, a personalized medicine framework that considers both host genetics and tumor biology may optimize the therapeutic potential of vitamin D in GI cancer prevention and management, providing a more targeted and rational approach than generalized supplementation strategies. The genetics and epigenetics of the effect of vitamin D are summarized in Table 3.

**Table 3:** Vitamin D and its molecular correlation

Topic	Key findings	Mechanisms	Clinical insights	Research gaps	References
Tumor-Specific Effects	- Vitamin D deficiency in up to 87.6% of GI cancer patients.	- Regulates proliferation, differentiation, apoptosis, and gut homeostasis.—Inhibits cancer stem cells.	- Supplementation reduced deficiency from 91.3% to 57.6%.—Shows promise in GI cancers (e.g., colorectal).	- Requires further studies to validate its role in prevention and treatment.—Long-term impact of supplementation remains unclear.	^[16,18,49,55]^
Genetic & Epigenetic Influences	- Polymorphisms in VDR and metabolizing enzymes linked to cancer risks (e.g., breast, prostate).	- VDR polymorphisms (e.g., Fok1 ff protective, GC rs7041 TT increases risk).	- Genetic factors may outweigh classical risk factors (e.g., sun exposure) for some cancers.	- Conflicting study results due to confounders like ethnicity and environment.—Need for large-scale genetic studies.	^[56–59]^
Precision Oncology Implications	- Vitamin D regulates cell processes (e.g., apoptosis, angiogenesis).	- Anti-cancer properties vary due to intratumor heterogeneity and individual variability.	- Biomarkers and genetic profiling may guide personalized therapies.—Limited success in clinical trials.	- High variability in responses to supplementation.—Insufficient understanding of mechanisms behind heterogeneity and supplementation outcomes.	^[53,60–62]^

## Vitamin D deficiency in high-risk populations

Vitamin D deficiency is disproportionately prevalent in high-risk populations due to a combination of environmental, physiological, and lifestyle factors. Postmenopausal women, individuals with inflammatory bowel disease (IBD), and corticosteroid-dependent asthmatics exhibit deficiency rates ranging from 60% to 90%, primarily due to impaired absorption, chronic inflammation, or medication-induced metabolic alterations^[63]^. In Arctic aboriginal populations, limited UVB exposure and the transition from traditional diets (rich in fatty fish and marine mammals) to Westernized foods have led to widespread deficiency^[64]^. Ethnic groups with increased skin melanin, such as individuals of Black and South Asian descent, face elevated risk due to reduced cutaneous synthesis of vitamin D^[65]^. Aging further exacerbates deficiency, with older adults showing decreased cutaneous production and limited dietary intake^[66]^.

In these populations, serum 25(OH)D levels often fall below the deficiency threshold of 20 ng/mL (50 nmol/L), contributing to adverse skeletal outcomes and potentially increasing cancer risk^[63,66]^. Among IBD patients, low vitamin D has been linked to increased disease activity, higher relapse rates, and elevated colorectal cancer (CRC) risk^[67,68]^. Mechanistically, 1,25-dihydroxyvitamin D_3_ regulates intestinal epithelial barrier integrity, suppresses pro-inflammatory cytokine production, and enhances antimicrobial peptide synthesis, thereby mitigating both inflammation and tumorigenesis^[69]^. Vitamin D also inhibits angiogenesis and epithelial-mesenchymal transition, while targeting cancer stem cell populations in CRC and other GI malignancies^[70]^. These mechanistic insights provide biological plausibility for the association between deficiency and disease progression, yet clinical translation remains complex.

Despite these promising mechanisms, establishing a causal role for vitamin D deficiency in IBD and CRC development remains challenging due to confounding factors such as dietary variation, comorbidities, and reverse causality^[67]^. Observational studies often report mixed results, likely influenced by variability in baseline vitamin D status, genetic polymorphisms in VDR and vitamin D metabolism genes, and differences in lifestyle or medication exposure. Large-scale longitudinal studies with standardized vitamin D measurements and adjustment for genetic and environmental factors are required to clarify the temporal and causal relationships between deficiency and disease outcomes.

Given the global prevalence of deficiency, estimated to affect over 1 billion people, public health strategies are urgently needed^[71]^. Recommended interventions include food fortification (e.g., dairy, cereals, oils), supplementation, and biofortification approaches^[72–74]^. Serum 25(OH)D levels of at least 75–80 nmol/L are associated with reduced risk of chronic diseases, including cancer^[73]^. Targeted screening and high-dose supplementation (e.g., 1000–4000 IU/day) may be necessary for high-risk groups, particularly those with limited sun exposure, chronic illness, or darker skin pigmentation^[66,73]^.

Due to its low cost, favorable safety profile, and broad systemic effects, improving vitamin D status in vulnerable populations represents a viable, evidence-supported strategy to enhance cancer prevention and reduce disease burden at the population level^[71,73]^. Integrating routine monitoring of 25(OH)D levels, personalized supplementation strategies based on risk stratification, and consideration of host genetic factors may further optimize preventive and therapeutic outcomes in these high-risk groups.

## Controversies and open questions

### Optimal serum levels of vitamin D

Determining the optimal serum 25-hydroxyvitamin D [25(OH)D] level for cancer prevention remains controversial due to variability in study populations, cancer types, and trial designs. Several studies associate levels ≥75 nmol/L (30 ng/mL) with improved bone health and reduced risk of falls, fractures, and colorectal cancer, whereas other evidence suggests that the most protective range may lie between 90 and 120 nmol/L (36–48 ng/mL)^[75–77]^. Conversely, some meta-analyses propose a U-shaped relationship, in which both low and excessively high levels are associated with increased risk, with an optimal cancer-protective window closer to 40–80 nmol/L (16–32 ng/mL)^[78]^. These discrepancies may arise from differences in baseline vitamin D status, genetic factors such as VDR polymorphisms, population-specific environmental exposures, and study endpoints (primary vs. secondary).

These conflicting thresholds highlight the need for personalized target ranges that account for age, comorbidities, baseline serum levels, genetic predispositions, and cancer type. For populations with low baseline vitamin D or high-risk features, supplementation may be necessary to reach protective thresholds. To maintain serum levels above 75 nmol/L, many experts recommend a daily intake of 1000–2000 IU, though intakes up to 4000 IU may be warranted in high-risk groups or those with limited UVB exposure^[75,76]^. Evidence from randomized trials and observational studies suggests that standardized monitoring of 25(OH)D and adjustment of dosing based on individual response may optimize preventive effects while minimizing risks associated with over-supplementation.

### Risks and safety of high-dose supplementation

While vitamin D supplementation is generally safe, concerns remain regarding over-supplementation. High-dose “stoss therapy” (e.g., single doses ≥100 000 IU or chronic intake >2800 IU/day) has been shown to normalize deficiency in up to 100% of patients^[79]^. However, long-term use may be associated with increased risk of hypercalcemia and hypercalciuria, even if kidney stone rates remain stable^[80]^.

Toxicity is rare but well-documented at doses exceeding 40,000 IU/day, with symptoms such as confusion, vomiting, and dehydration due to severe hypercalcemia^[81,82]^. Although the no observed adverse effect level (NOAEL) is currently set at 2000 IU/day, many experts argue that this is overly conservative, especially for deficient or high-risk populations^[82]^. A daily intake of up to 4000 IU/day is generally considered safe for maintaining serum levels >100 nmol/L in such individuals.

### Sunlight Exposure vs. Oral supplementation

Ultraviolet B (UVB) exposure remains the most natural and efficient source of vitamin D synthesis, yet it poses a paradox: while promoting vitamin D production, it also increases the risk of skin cancers, particularly with chronic or high-dose exposure^[83,84]^. Even brief, unprotected UVB exposure can cause DNA mutations leading to melanoma or squamous cell carcinoma.

At the same time, observational studies show that higher serum vitamin D levels, regardless of source, are associated with reduced colorectal cancer risk and all-cause mortality^[85,86]^. This underscores the challenge of balancing sun exposure with cancer prevention efforts.

Clinical guidance should favor short, controlled sun exposure (e.g., 10–15 minutes on arms/legs, 3 times per week) in low-risk individuals, while relying on oral supplementation in people at higher risk for skin cancer, those living at high latitudes, or with limited outdoor activity^[86]^. Public health messaging must address both sides of the UV-vitamin D paradox, ensuring that sun protection campaigns do not inadvertently exacerbate deficiency-related health risks^[85,86]^.

## Future directions and recommendations

Advancing the understanding of vitamin D’s role in gastrointestinal (GI) cancers requires a coordinated, multidisciplinary research agenda. Despite promising observational data and biological plausibility, current evidence from randomized trials remains inconclusive, partly due to methodological variability and population heterogeneity.

### Mechanistic and molecular research

Future studies should further explore vitamin D’s regulation of inflammatory pathways (e.g., cytokine cascades, NF-κB signaling), tumor suppression mechanisms, and its role in modulating the tumor microenvironment^[87,88]^. Calcitriol’s role in maintaining epithelial barrier integrity and mucosal immunity offers a promising avenue for GI-specific cancer prevention strategies^[16]^.

Genome-wide studies of vitamin D receptor (VDR) target genes, as well as single-cell transcriptomic analyses of vitamin D-treated tumor tissues, may reveal differential responses across molecular subtypes. Additionally, the interplay between vitamin D signaling and gut microbiota requires deeper investigation to determine whether microbiome-mediated effects contribute to cancer outcomes.

### Clinical and interventional research

There is an urgent need for well-powered, multicenter randomized controlled trials (RCTs) that assess vitamin D supplementation in defined subpopulations, such as individuals with genetic polymorphisms affecting vitamin D metabolism, or those with precancerous GI lesions. Trials should incorporate stratification by baseline serum levels, BMI, comorbidities, and race/ethnicity to identify responders vs. non-responders.

Real-world data from longitudinal cohorts, registries, and electronic health records could supplement RCT findings by providing insight into dose-response relationships and long-term safety outcomes. Trials assessing the synergistic effects of vitamin D with immunotherapy or chemoprevention also represent a promising frontier^[88]^.

### Implementation, monitoring, and public health strategy

Vitamin D deficiency is common among cancer patients and the general population^[89,90]^, yet routine screening and supplementation are not uniformly implemented. Expert consensus recommends maintaining serum 25(OH)D levels ≥30 ng/mL for skeletal and possibly oncologic benefits^[91,92]^. However, no standardized dosing or monitoring protocols exist across cancer centers.

High-risk groups, including the elderly, those with chronic GI diseases, and individuals with darker skin, may benefit from tailored supplementation plans, beginning with baseline 25(OH)D assessment and follow-up testing every 3–6 months. Suggested daily doses range from 800–1000 IU for maintenance, up to 2000–4000 IU in deficient states^[90–92]^.

Policy-level approaches such as food fortification, supplementation programs, and public awareness campaigns can help address population-wide deficiency, particularly in regions with low UVB exposure.

### Conceptual framework and open questions

The DINOMIT model offers a conceptual pathway for vitamin D’s role in cancer development: Disjunction → Initiation → Natural selection → Overgrowth → Metastasis → Involution → Transition^[93,94]^. Research should assess at which phases vitamin D intervention is most effective, particularly in GI cancers.

Key unanswered questions include:

- What is the optimal serum 25(OH)D range for cancer prevention vs. improved survival?
- Which patient subgroups are most likely to benefit from supplementation?
- How do genetic, epigenetic, and microbiome-related factors influence individual responses?
- Can vitamin D act synergistically with other anticancer agents?

## Clinical implications

The current body of evidence suggests that vitamin D may have a meaningful, yet context-dependent, role in gastrointestinal (GI) cancer prevention and management. While preclinical and mechanistic studies demonstrate anticancer effects via VDR-mediated regulation of proliferation, apoptosis, immune modulation, and gut microbiota homeostasis, clinical translation remains limited by inconsistent trial outcomes, population heterogeneity, and variable baseline vitamin D status. Given the high prevalence of vitamin D deficiency among GI cancer patients and high-risk populations (e.g., older adults, postmenopausal women, individuals with inflammatory bowel disease, and populations with darker skin), routine assessment of serum 25-hydroxyvitamin D [25(OH)D] levels may be considered, particularly in patients with known risk factors. Baseline measurement allows identification of deficiency (<20 ng/mL or 50 nmol/L) and enables tailored supplementation strategies. For individuals with documented deficiency, supplementation is generally safe and can restore optimal serum levels. Standard maintenance dosing ranges from 800 to 1000 IU/day, while higher doses (2000–4000 IU/day) may be warranted for deficient or high-risk individuals. Personalized dosing strategies should consider baseline 25(OH)D, BMI, comorbidities, UVB exposure, and potential genetic factors (e.g., VDR polymorphisms) that influence vitamin D metabolism and responsiveness. Evidence from RCTs and meta-analyses suggests a potential role for vitamin D supplementation in improving cancer prognosis, particularly in colorectal cancer, although findings are not yet conclusive. Clinicians may consider supplementation as an adjunctive measure alongside standard therapies (e.g., surgery, chemotherapy, immunotherapy), especially in patients with low baseline vitamin D levels. However, routine use solely for cancer prevention is not currently supported by definitive clinical trial data. Vitamin D supplementation is well-tolerated within recommended limits, with toxicity being rare and usually associated with excessive dosing (>40,000 IU/day). Periodic monitoring of serum 25(OH)D and calcium levels is advisable in patients receiving higher doses or with predisposing conditions for hypercalcemia. Clinicians should adopt a pragmatic, risk-stratified approach:

- High-risk or deficient patients: initiate supplementation with follow-up monitoring to achieve target serum levels (~30–50 ng/mL).
- Patients with sufficient baseline levels: routine supplementation for cancer prevention may be optional, emphasizing lifestyle measures such as safe sun exposure and dietary intake.
- Integration with multidisciplinary care: vitamin D status may be considered alongside genetic, dietary, and microbiome factors to optimize individualized prevention and therapeutic strategies.

## Conclusion

Vitamin D plays a multifaceted role in cancer biology, offering potential benefits in prevention, prognosis, and treatment, particularly for gastrointestinal (GI) cancers. Mechanistically, vitamin D and its receptor (VDR) regulate cell proliferation, differentiation, apoptosis, immune modulation, and gut microbiota, all of which are crucial for maintaining tissue homeostasis and combating cancer progression. Epidemiological evidence supports an inverse association between vitamin D levels and cancer incidence, though clinical trials have yielded mixed results, highlighting the complexity of its role.

While vitamin D deficiency is prevalent among high-risk populations, supplementation shows promise in reducing cancer-related mortality and improving outcomes in certain cases. However, challenges such as variability in individual responses, genetic and epigenetic influences, and optimal dosing regimens underscore the need for personalized approaches and further research. Addressing confounding factors in observational studies and refining interventional strategies will be key to unlocking the full potential of vitamin D in cancer prevention and therapy.

## Data Availability

Not applicable for this review.

## Peer and provenance statement

Not commissioned, externally peer-reviewed.
